# Long‐Term Health Outcomes in Individuals With Confirmed Versus Unconfirmed Undiagnosed Diabetes Based on Oral Glucose Tolerance Test: Findings From the Tehran Lipid and Glucose Study

**DOI:** 10.1155/jdr/4228661

**Published:** 2026-07-08

**Authors:** Navid Ebrahimi, Samaneh Asgari, Maryam Tohidi, Davood Khalili, Fereidoun Azizi, Sepideh Besarat, Farzad Hadaegh

**Affiliations:** ^1^ Prevention of Metabolic Disorders Research Center, Research Institute for Metabolic and Obesity Disorders, Research Institute for Endocrine Sciences, Shahid Beheshti University of Medical Sciences, Tehran, Iran, sbmu.ac.ir; ^2^ Endocrine Research Center, Research Institute for Endocrine Sciences, Shahid Beheshti University of Medical Sciences, Tehran, Iran, sbmu.ac.ir

**Keywords:** cardiovascular diseases, glucose tolerance test, longitudinal study, type 2 diabetes mellitus

## Abstract

**Background:**

To evaluate the prognostic performance of the single‐sample confirmatory definition of undiagnosed diabetes on outcomes, including hypertension, chronic kidney disease (CKD), cardiovascular disease (CVD), and mortality, in the Tehran Lipid and Glucose Study cohort.

**Methods:**

Among 6712 participants (3682 women) aged ≥ 30 years not on glucose‐lowering medication, unconfirmed undiagnosed diabetes was defined as either fasting plasma glucose (FPG) ≥ 7 mmol/L or 2‐h postload glucose (2‐h PG) ≥ 11.1 mmol/L, whereas confirmed undiagnosed diabetes required both. Multivariable Cox models estimated hazard ratios (HRs) and 95% confidence intervals (CIs) for each outcome.

**Results:**

At baseline, 3.6% and 3.3% of participants had confirmed or unconfirmed undiagnosed diabetes, respectively. During a median follow‐up of over 10 years, both unconfirmed and confirmed undiagnosed diabetes were associated with a higher risk of developing treated diabetes, with HRs (95% CI) of 7.73 (6.35–9.44) and 17.30 (14.32–20.83), respectively. Confirmed undiagnosed diabetes was further associated with incident CKD (1.33 [1.02–1.73]), hypertension (1.26 [1.00–1.60]), first CVD (1.40 [1.01–1.93]), and hard CVD (1.65 [1.04–2.61]), whereas the association with all‐cause mortality did not reach statistical significance (1.50 [0.96–2.33], *p* = 0.07). Unconfirmed undiagnosed diabetes was only associated with treated diabetes and first CVD (1.53 [1.11–2.12]). Isolated FPG and 2‐h PG elevations showed similar risks for treated diabetes (9.00 [6.26–13.00] and 7.40 [5.92–9.24], respectively), but only isolated FPG was significantly associated with first CVD (2.55 [1.50–4.31]) and hard CVD (2.43 [1.06–6.00]), comparable with confirmed undiagnosed diabetes.

**Conclusions:**

Confirmed undiagnosed diabetes was associated with a higher risk of cardiovascular and renal outcomes. The unconfirmed undiagnosed phenotype shared a similar CVD risk with confirmed diabetes but had a 50% lower risk of treated diabetes, with a stronger association observed for isolated FPG elevation than for isolated 2‐h PG.

## 1. Introduction

The global burden of diabetes continues to rise, with prevalence increasing from 3.2% in 1990 to 6.1% in 2021 and projected to reach 9.6% by 2050 [1]. The Middle East and North Africa (MENA) region has the highest age‐standardized prevalence (9.3% in 2021), expected to reach 16.8% by 2050, along with a substantial burden of disability‐adjusted life years (DALYs), largely driven by Type 2 diabetes mellitus (T2DM) [1, 2]. Approximately one‐third of cases in this region remain undiagnosed, particularly in low‐ and middle‐income countries. In Iran, the incidence of T2DM is high, estimated at 36.3 per 1000 person‐years, corresponding to over 800,000 new cases annually [3].

Current diagnostic criteria emphasize hemoglobin A1c (HbA1c) as a practical indicator of long‐term glycemia, reducing reliance on the oral glucose tolerance test (OGTT), except in specific settings such as gestational diabetes [4, 5]. In the absence of classic symptoms, diabetes can be diagnosed using fasting plasma glucose (FPG), 2‐h postload glucose (2‐h PG), or HbA1c, provided that two abnormal results are confirmed either concurrently or at separate time points [5]. Given the often‐prolonged asymptomatic phase of hyperglycemia, early detection is essential, as undiagnosed diabetes is associated with an increased risk of complications [6].

Guidelines recommend confirming an isolated abnormal glucose value with a second test, whereas concurrent abnormalities in two indices are sufficient for diagnosis [5]. Some studies, however, have used single‐sample definitions to estimate undiagnosed diabetes [7, 8]. Data from the National Health and Nutrition Examination Survey (NHANES) show a decline in confirmed undiagnosed diabetes in US adults from 19.3% in 1988 to 9.5% in 2020 [8]. In a 25‐year US cohort, both confirmed and unconfirmed undiagnosed diabetes, based on FPG and HbA1c, were associated with higher cardiovascular disease (CVD) risk (52% and 20%, respectively) compared with individuals without diabetes [9]. In contrast, a UK population‐based study with repeated OGTT and HbA1c measurements found that only HbA1c‐confirmed cases had increased risks of CVD and chronic kidney disease (CKD), whereas unconfirmed cases had risks similar to those without diabetes [10].

Despite the high burden of undiagnosed diabetes in the MENA region, evidence on the prognostic significance of confirmed versus unconfirmed undiagnosed diabetes is limited. In Iran, a national STEPS survey in 2016 reported a 2.7% prevalence of undiagnosed diabetes based on elevated FPG alone [11]. The present study is aimed at evaluating the prognostic performance of a single‐sample confirmatory definition of undiagnosed diabetes. We examined the association between baseline diabetes status and subsequent outcomes, including hypertension, CKD, CVD events, and all‐cause mortality, and assessed the independent effects of isolated elevations in FPG and 2‐h PG.

## 2. Materials and Methods

### 2.1. Study Design

The Tehran Lipid and Glucose Study (TLGS) is a community‐based prospective cohort established on a representative sample of residents of District 13 of Tehran; in Phases 1 and 2, participants were recruited using multistage cluster random sampling and followed at approximately 3‐year intervals [12, 13]. As previously reported, the cohort′s age distribution and socioeconomic characteristics were broadly representative of the Tehran population at the time of study initiation [12, 13]. Detailed information on the design and methodology of the TLGS has been published previously [12]. For the present analysis, we included 7241 participants aged ≥ 30 years who attended Phase 2. The study was approved by the institutional review board of the Research Institute for Endocrine Sciences, Shahid Beheshti University of Medical Sciences (IR.SBMU.ENDOCRINE.REC.1404.041), and all participants provided written informed consent.

### 2.2. Study Population

**Figure 1:**
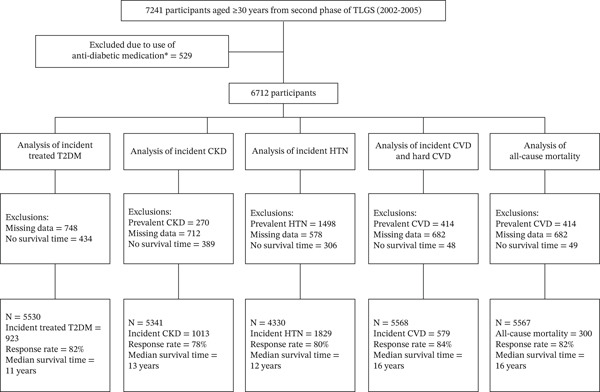
Flowchart of the study population, Tehran Lipid and Glucose Study, 2002–2020. ∗Participants using diabetes medications were excluded from the final analysis due to their small number.

Figure 1 shows participant selection for each outcome. Of 7241 Phase 2 participants aged ≥ 30 years, we excluded 529 who were using glucose‐lowering medication at baseline, leaving 6712 eligible individuals (3682 women). Outcome‐specific exclusions for prevalent disease, missing covariates, and no follow‐up time yielded analytic samples of 5530 for treated diabetes, 4330 for hypertension, 5341 for CKD, 5568 for CVD, and 5567 for all‐cause mortality. Median follow‐up ranged from 11 years for treated diabetes to 16 years for CVD and mortality. Figure S1 shows the selection process for the imputed dataset used in the sensitivity analyses addressing missing data.

### 2.3. Clinical and Laboratory Measurements

Baseline clinical and laboratory measurements were obtained using standardized TLGS protocols. Information on demographics, education, medication use, smoking, and family history was collected by questionnaire. Weight was measured with participants in light clothing using a digital scale. Height was measured using a tape meter in a standing position without shoes while shoulders were in normal alignment. Waist circumference (WC) was measured at the level of the umbilicus. Body mass index (BMI) was calculated as weight in kilograms divided by height in square meters (kg/m^2^). Systolic and diastolic blood pressure (SBP and DBP, respectively) were measured twice on the right arm, with the average used for analysis, after participants had rested for 15 min, using a mercury sphygmomanometer. Blood samples were taken after 12–14 h of fasting and analyzed on the same day at the TLGS research laboratory using Pars Azmoon kits (Pars Azmoon Inc., Tehran, Iran) and a Selectra 2 autoanalyzer (Vital Scientific, Spankeren, the Netherlands). For participants not on glucose‐lowering medications, 2‐h PG was measured following a standard OGTT. Glucose concentrations were assessed using an enzymatic colorimetric method with glucose oxidase. The glomerular filtration rate (eGFR) was estimated using the updated 2021 equation developed by the Chronic Kidney Disease Epidemiology Collaboration (CKD‐EPI) [14]. Additional laboratory measurements, including FPG, 2‐h PG, triglycerides (TG), total cholesterol (TC), high‐density lipoprotein cholesterol (HDL‐C), and creatinine, have been previously described in detail [13].

### 2.4. Definition of Diabetes Phenotypes

We defined the “no diabetes” as FPG levels < 7 mmol/L (126 mg/dL) and 2‐h PG < 11.1 mmol/L (< 200 mg/dL). Unconfirmed undiagnosed diabetes was defined by elevated levels of either FPG ≥7 mmol/L (126 mg/dL) or 2‐h PG ≥ 11.1 mmol/L (≥ 200 mg/dL), but not both, whereas confirmed undiagnosed diabetes was defined as having elevated levels of both FPG ≥7 mmol/L (126 mg/dL) and 2‐h PG ≥ 11.1 mmol/L (≥ 200 mg/dL). Individuals with isolated FPG elevation and isolated 2‐h PG elevation were defined as having only one of these conditions. Participants who reported the use of glucose‐lowering medications during the follow‐up were considered as “treated diabetes” cases.

### 2.5. Definition of Terms and Outcomes

Education levels were divided into three categories: less than 6 years, 6–12 years, and more than 12 years. Current smokers were defined as individuals using any form of tobacco (cigarettes, pipes, or water pipes) at the time of the examination. A positive family history of diabetes was defined as having a first‐degree relative diagnosed with diabetes. A positive family history of premature CVD was defined as coronary heart disease (CHD) and/or stroke in a first‐degree relative before the ages of 55 years for male relatives and 65 years for female relatives. Hypertension was defined as a SBP of ≥ 140 mmHg, DBP of ≥ 90 mmHg, or the use of antihypertensive medications. CKD was defined as eGFR of less than 60 mL/min/1.73 m^2^ and a decline in eGFR of ≥ 25% from baseline during the follow‐up period [15].

Details regarding cardiovascular outcomes have been previously described [16]. In the TLGS study, participants were monitored annually for any medical events leading to hospitalization through telephone follow‐ups. A trained nurse initially inquired about medical conditions, followed by a trained physician who gathered additional information during a visit and by reviewing medical records. When necessary, an outcome assessment committee, composed of an internist, endocrinologist, cardiologist, epidemiologist, pathologist, and other specialists, evaluated the data to determine specific outcomes for each event. CVD was coded based on the 10th Revision of the International Classification of Diseases (ICD‐10) and categorized into two groups: first incident CVD and hard CVD events. Incident CVD events were defined as definite myocardial infarction (MI), probable MI, unstable angina, angiographic‐proven CHD, and stroke. First hard CVD was defined as a composite of CVD‐related death, nonfatal MI, or nonfatal stroke. In case of mortality, data were collected either from hospitals by authorized local physicians or from death certificates and were subsequently evaluated by the TLGS outcome committee.

### 2.6. Statistical Analysis

Baseline characteristics of the study population were summarized as mean (standard deviation [SD]) for normally distributed continuous variables, median (interquartile range [IQR]) for nonnormally distributed variables (e.g., TG, FPG, and 2‐h PG), and count (percentage) for categorical variables. The normality of continuous variables was assessed using the Shapiro–Wilk test. Characteristics were described by diabetes status and in the total population for the outcome with the largest sample size (i.e., incident CVD). The comparison of baseline characteristics between confirmed and unconfirmed undiagnosed diabetes was performed using Student′s *t*‐test for normally distributed continuous variables, the Kruskal–Wallis test for skewed or ordinal variables, and the chi‐square test for categorical variables. Moreover, a post hoc pairwise comparison between confirmed and unconfirmed undiagnosed diabetes was performed for each variable separately.

To evaluate the association of diabetes status categories with the incident of each outcome, Cox proportional hazard models were applied; Model 1 was adjusted with age and sex; Model 2 was further adjusted with BMI, family history of diabetes, family history of premature CVD, education levels (< 6 years as reference), current smoking, lipid medications, non–HDL‐C, hypertension (except for incident hypertension), SBP (only for incident hypertension), and eGFR. The adjusted hazard ratios (HRs) and 95% confidence intervals (CIs) were reported for each of the diabetes status categories, considering “no diabetes” as the reference. The event date for incident T2DM, hypertension, and CKD cases was described as the midtime between the date of the follow‐up visit at which each outcome was detected for the first time and the most recent follow‐up visit preceding the diagnosis; the follow‐up time was drawn from the difference between the calculated midtime date and the date at which the subjects entered the study. For the censored and lost follow‐up individuals, the survival time was the interval between the first and the last observation dates (i.e., the last follow‐up time for incident hypertension, CKD, and known diabetes was set until March 2022, whereas for CVD/mortality events, it was determined as of March 2018.). The proportionality in the Cox models was evaluated with the Schoenfeld residual test; generally, all proportionality assumptions were appropriate.

To formally compare the magnitude of associations between confirmed and unconfirmed undiagnosed diabetes across each clinical outcome, we used the Wald test. A two‐tailed *p* value of < 0.05 was considered to indicate a statistically significant difference between the HRs.

We examined gender interactions across diabetes status categories for six outcomes, applying Bonferroni correction and setting the significance threshold at 0.008 (0.05/6). All interaction *p* values exceeded this threshold. As a sensitivity analysis, we handled missing data using multiple imputation by chained equations (MICE), performing 15 imputations to reflect the highest proportion of missingness (about 15%) among variables [17].

All analyses were conducted using STATA Version 17 SE (StataCorp, Texas, United States), and a two‐tailed *p* value of < 0.05 was considered significant.

## 3. Results

### 3.1. Baseline Characteristics

Baseline characteristics are shown in Table 1. In the CVD analytic sample (*n* = 5568; 55.1% women; mean age: 47.5 years), 3.6% had confirmed and 3.3% had unconfirmed undiagnosed diabetes. Compared with participants without diabetes, both groups had higher BMI and blood pressure, lower eGFR, and lower educational attainment. Compared with the unconfirmed group, the confirmed group had higher FPG, 2‐h PG, TC, and TG. When comparing the two groups of confirmed and unconfirmed undiagnosed diabetes, the mean levels of FPG and 2‐h PG were significantly higher in the confirmed undiagnosed group than in the unconfirmed group (both *p* < 0.001). Individuals with confirmed undiagnosed diabetes also had significantly higher levels of TC and TG than those with unconfirmed undiagnosed diabetes (both *p* < 0.05).

**Table 1:** Baseline characteristics of TLGS participants by diabetes status (2002–2005) (*N* = 5568^a^).

	Total population (*N* = 5568)	No diabetes (*N* = 5180)	Confirmed undiagnosed diabetes (*N* = 203)	Unconfirmed undiagnosed diabetes (*N* = 185)	*p* ^b^	*p* ^c^
Sex (female %)	3070 (55.1)	2857 (55.2)	107 (52.7)	106 (57.3)	0.66	0.36
Age, years	47.53 (12.40)	47.04 (12.33)	54.63 (11.21)	53.53 (11.94)	**< 0.001**	0.65
BMI, kg/m^2^	28.00 (4.59)	27.84 (4.58)	29.82 (4.14)	29.86 (4.54)	**< 0.001**	1.00
WC, cm	93.21 (11.40)	92.70 (11.36)	100.47 (9.45)	99.53 (9.88)	**< 0.001**	0.69
SBP, mmHg	117.69 (18.35)	116.79 (17.84)	130.67 (22.60)	128.68 (18.40)	**< 0.001**	0.52
DBP, mmHg	75.67 (10.43)	75.42 (10.40)	78.90 (10.53)	79.12 (10.15)	**< 0.001**	0.98
FPG, mmol/L	5.1 (4.7–5.4)	5.0 (4.7–5.3)	8.4 (7.6–10.1)	6.4 (5.9–6.9)	**< 0.001**	**< 0.001**
2‐h PG, mmol/L	6.1 (5.0–7.3)	5.9 (4.9–7.0)	17.3 (14.3–20.6)	12.0 (11.2–13.2)	**< 0.001**	**< 0.001**
HDL‐C, mmol/L	1.01) 0.26)	1.01 (0.26)	0.96 (0.24)	0.97 (0.23)	**0.036**	0.94
TC, mmol/L	5.11 (1.03)	5.07 (1.02)	5.74 (1.09)	5.40 (1.04)	**< 0.001**	**0.003**
TG, mmol/L	1.62 (1.13–2.29)	1.57 (1.11–2.21)	2.38 (1.80–3.39)	2.17 (1.54–2.98)	**< 0.001**	**0.002**
eGFR, mL/min/1.73 m^2^	82.85 (14.34)	83.14 (14.29)	79.06 (14.46)	78.62 (14.61)	**< 0.001**	0.95
Education level					**< 0.001**	0.57
< 6 years	1899 (34.1)	1683 (32.4)	115 (56.6)	101 (54.5)		
≥ 6 to < 12 years	2839 (51.0)	2703 (52.1)	67 (33.0)	69 (37.3)		
≥ 12 years	830 (14.9)	794 (15.3)	21 (10.3)	15 (8.1)		
Family history of diabetes	1051 (18.9)	938 (18.1)	57 (28.0)	56 (30.2)	**< 0.001**	0.63
Family history of premature CVD (%)	374 (6.7)	352 (6.8)	10 (4.9)	12 (6.5)	0.58	0.66
Current smoker (%)	1009 (18.1)	948 (18.3)	33 (16.2)	28 (15.1)	0.43	0.76
Hypertension (%)	2341 (42.0)	2094 (40.4)	123 (60.5)	124 (67)	**< 0.001**	0.19
Lipid lowering medication use (%)	138 (2.5)	124 (2.3)	4 (1.9)	10 (5.4)	**0.031**	0.07

*Note:* Values are shown as mean (SD) and number (%) for continuous and categorical variables, respectively, and median (interquartile range) for TG, FPG, and 2‐h PG. No diabetes: FPG < 7 mmol/L and 2‐h PG < 11 mmol/L among participants without diagnosed diabetes. Confirmed undiagnosed diabetes: elevated levels of both FPG (≥ 7 mmol/L) and 2‐h PG (≥ 11 mmol/L) among participants without diagnosed diabetes. Unconfirmed undiagnosed diabetes: elevated levels of FPG (≥ 7 mmol/L) or 2‐h PG (≥ 11 mmol/L), but not both, among participants without diagnosed diabetes. The bolded values in indicate statistically significant differences between the compared groups (*p* < 0.05).

Abbreviations: 2‐h PG, 2‐h postload plasma glucose; BMI, body mass index; DBP, diastolic blood pressure; eGFR, estimated glomerular filtration rate; FPG, fasting plasma glucose; HDL‐C, high‐density lipoprotein cholesterol; SBP, systolic blood pressure; TC, total cholesterol; TG, triglycerides; WC, waist circumference.

^a^The study population for the CVD outcome, which had the largest sample size among the different outcomes.

^b^ *p* value for comparisons among the three groups. The comparison *p* value between groups was calculated using the ANOVA test for normal continuous variables, the Kruskal–Wallis test for skewed variables, and the chi‐square test for categorical variables.

^c^ *p* value for the comparison between confirmed and unconfirmed undiagnosed diabetes (post hoc test).

### 3.2. Association of Different Phenotypes of Diabetes With Clinical Outcomes

The HRs and 95% CIs for the defined clinical outcomes were shown in Table 2. Compared with individuals without diabetes, those with unconfirmed undiagnosed and confirmed undiagnosed diabetes had a significantly higher risk of developing treated diabetes, with HRs of 7.73 (95% CI: 6.35–9.44) and 17.30 (95% CI: 14.32–20.83), respectively. The comparison of HRs between the confirmed and unconfirmed undiagnosed groups yielded a *p* value of < 0.001 based on the Wald test.

**Table 2:** Multivariable hazard ratios (HRs) and 95% confidence intervals (CIs) for clinical outcomes by baseline diabetes phenotype at TLGS study (2002–2005).

	E/N	Model 1		Model 2	
		HR (95% CI)	*p*	HR (95% CI)	*p*
Incident treated diabetes					
No diabetes	614/5134	Reference		Reference	
Unconfirmed undiagnosed	129/194	**9.23 (7.58–11.22)**	**< 0.001**	**7.73 (6.35–9.44)**	**< 0.001**
Confirmed undiagnosed	180/202	**24.80 (20.74–29.65)**	**< 0.001**	**17.3 (14.32–20.83)** ^a^	**<0.001**
Incident CKD					
No diabetes	899/4970	Reference		Reference	
Unconfirmed undiagnosed	52/182	1.28 (0.97–1.69)	0.09	1.18 (0.89–1.57)	0.24
Confirmed undiagnosed	62/189	**1.35 (1.04–1.75)**	**0.02**	**1.33 (1.02–1.73)**	**0.03**
Incident hypertension					
No diabetes	1676/4093	Reference		Reference	
Unconfirmed undiagnosed	75/115	**1.61 (1.27–2.03)**	**< 0.001**	**1.26 (1.00–1.60)**	**0.048**
Confirmed undiagnosed	78/122	**1.62 (1.29–2.04)**	**< 0.001**	1.13 (0.90–1.43)	0.29
Incident first CVD					
No diabetes	496/5180	Reference		Reference	
Unconfirmed undiagnosed	41/185	**1.79 (1.30–2.47)**	**< 0.001**	**1.53 (1.11–2.12)**	**0.01**
Confirmed undiagnosed	42/203	**1.70 (1.24–2.33)**	**0.001**	**1.40 (1.01–1.93)** ^b^	**0.04**
Incident hard CVD					
No diabetes	203/5180	Reference		Reference	
Unconfirmed undiagnosed	16/185	1.57 (0.94–2.61)	0.08	1.27 (0.76–2.13)	0.36
Confirmed undiagnosed	21/203	**1.93 (1.23–3.03)**	**0.004**	**1.65 (1.04–2.61)**	**0.03**
All‐cause mortality					
No diabetes	262/5180	Reference		Reference	
Unconfirmed undiagnosed	16/184	1.14 (0.69–1.89)	0.60	1.09 (0.66–1.83)	0.73
Confirmed undiagnosed	22/203	1.45 (0.94–2.25)	0.09	1.50 (0.96–2.33)	0.07

*Note:* Model 1: adjusted for age and sex. Model 2: adjusted for age, sex, BMI, family history of diabetes, family history of CVD, education levels, current smoking, lipid medications, non–HDL‐C, hypertension (except for incident hypertension), SBP (only for incident hypertension), and eGFR. E/N may differ across outcomes due to outcome‐specific eligibility and missing data on covariates/outcomes. No diabetes: FPG < 7 mmol/L and 2‐h PG < 11 mmol/L among participants without diagnosed diabetes. Confirmed undiagnosed diabetes: elevated levels of both FPG (≥ 7 mmol/L) and 2‐h PG (≥ 11 mmol/L) among participants without diagnosed diabetes. Unconfirmed undiagnosed diabetes: elevated levels of FPG (≥ 7 mmol/L) or 2‐h PG (≥ 11 mmol/L), but not both, among participants without diagnosed diabetes. Boldfaced hazard ratios (HRs) indicate statistically significant associations (*p* < 0.05).

Abbreviations: CI, confidence interval; CKD, chronic kidney disease; CVD, cardiovascular disease; E, events; eGFR, estimated glomerular filtration rate; HDL‐C, high‐density lipoprotein cholesterol; HR, hazard ratio; N, number; SBP, systolic blood pressure.

^a^Incident treated diabetes: The *p* value of HR comparison of confirmed undiagnosed to unconfirmed undiagnosed was < 0.001, applying Wald test.

^b^Incident first CVD: The *p* value of HR comparison of confirmed undiagnosed to unconfirmed undiagnosed was 0.704, applying the Wald test.

For incident CKD, participants with confirmed undiagnosed diabetes had a significantly higher risk (HR = 1.33, 95% CI: 1.02–1.73, *p* = 0.03), whereas no significant association was observed among those with unconfirmed undiagnosed diabetes (HR = 1.18, 95% CI: 0.89–1.57, *p* = 0.24).

For incident hypertension, participants with unconfirmed undiagnosed diabetes had a significantly higher risk (HR = 1.26, 95% CI: 1.00–1.60, *p* = 0.048), whereas the association among those with confirmed undiagnosed diabetes was not significant (HR = 1.13, 95% CI: 0.90–1.43, *p* = 0.29).

Both unconfirmed and confirmed undiagnosed diabetes were significantly associated with an increased risk of a first CVD event. The HR for unconfirmed undiagnosed diabetes was 1.53 (95% CI: 1.11–2.12, *p* = 0.01), whereas that for confirmed undiagnosed diabetes was 1.40 (95% CI: 1.01–1.93, *p* = 0.04). There was no significant difference between these HRs based on the Wald test (*p* = 0.70).

For hard CVD events, individuals with unconfirmed undiagnosed diabetes had a 27% higher risk that did not reach statistical significance (HR = 1.27, 95% CI: 0.76–2.13, *p* = 0.36), whereas those with confirmed undiagnosed diabetes had a significantly higher risk of developing a hard CVD event (HR = 1.65, 95% CI: 1.04–2.61, *p* = 0.03).

For all‐cause mortality, no significant associations were observed for either unconfirmed or confirmed undiagnosed diabetes. The HRs were 1.09 (95% CI: 0.66–1.83, *p* = 0.73) and 1.50 (95% CI: 0.96–2.33, *p* = 0.07), respectively.

### 3.3. Association of Isolated FPG and 2‐h PG Elevations With Clinical Outcomes

Table 3 presents the HRs and CIs for the associations between isolated elevation of FPG and isolated elevation of 2‐h PG elevation with various clinical outcomes.

**Table 3:** Multivariable hazard ratios (HRs) and 95% confidence intervals (CIs) for clinical outcomes by confirmed undiagnosed diabetes and isolated elevation of FPG and 2‐h PG, Tehran Lipid and Glucose Study (2002–2005).

	E/N	Model 1		Model 2	
		HR (95% CI)	*p*	HR (95% CI)	*p*
Incident treated diabetes					
No diabetes	614/5134	Reference		Reference	
Confirmed undiagnosed	180/202	**24.77 (20.72–29.61)**	**< 0.001**	**17.29 (14.34–20.85)**	**< 0.001**
Isolated elevation of FPG	32/40	**13.19 (9.22–18.86)**	**< 0.001**	**9.00 (6.26–13.00)**	**< 0.001**
Isolated elevation of 2‐h PG	97/154	**8.37 (6.72–10.43)**	**< 0.001**	**7.40 (5.92–9.24)**	**< 0.001**
Incident CKD					
No diabetes	899/4970	Reference		Reference	
Confirmed undiagnosed	62/189	**1.35 (1.04–1.75)**	**0.02**	**1.33 (1.02–1.73)**	**0.03**
Isolated elevation of FPG	13/37	1.67 (0.97–2.90)	0.06	1.37 (0.79–2.39)	0.26
Isolated elevation of 2‐h PG	39/145	1.18 (0.86–1.63)	0.30	1.13 (0.82–1.57)	0.45
Incident hypertension					
No diabetes	1676/4093	Reference		Reference	
Confirmed undiagnosed	78/122	**1.62 (1.29–2.04)**	**< 0.001**	1.13 (0.90–1.43)	0.29
Isolated elevation of FPG	15/24	**2.12 (1.27–3.52)**	**0.004**	1.56 (0.93–2.60)	0.09
Isolated elevation of 2‐h PG	60/91	**1.52 (1.17–1.97)**	**0.002**	1.21 (0.93–1.57)	0.15
Incident first CVD					
No diabetes	496/5180	Reference		Reference	
Confirmed undiagnosed	42/203	**1.70 (1.24–2.33)**	**0.001**	**1.40 (1.01–1.93)**	**0.04**
Isolated elevation of FPG	15/40	**3.54 (2.11–5.92)**	**< 0.001**	**2.55 (1.50–4.31)**	**0.001**
Isolated elevation of 2‐h PG	26/145	1.39 (0.94–2.07)	0.1	1.23 (0.82–1.84)	0.30
Incident hard CVD					
No diabetes	203/5180	Reference		Reference	
Confirmed undiagnosed	21/203	**1.93 (1.23–3.03)**	**0.004**	**1.65 (1.04–2.61)**	**0.03**
Isolated elevation of FPG	6/40	**3.50 (1.55–7.90)**	**0.003**	**2.43 (1.06–6.00)**	**0.04**
Isolated elevation of 2‐h PG	10/145	1.18 (0.62–2.22)	0.61	1.00 (0.52–1.89)	0.99
All‐cause mortality					
No diabetes	262/5180	Reference		Reference	
Confirmed undiagnosed	22/203	1.45 (0.94–2.25)	0.09	1.50 (0.96–2.33)	0.07
Isolated elevation of FPG	4/39	1.68 (0.62–4.52)	0.30	1.63 (0.60–4.46)	0.34
Isolated elevation of 2‐h PG	12/145	1.03 (0.58–1.84)	0.91	1.00 (0.55–1.77)	0.97

*Note:* Model 1: adjusted for age and sex. Model 2: adjusted for age, sex, BMI, family history of diabetes, family history of CVD, education levels, current smoking, lipid medications, non–HDL‐C, hypertension (except for incident hypertension), SBP (only for incident hypertension), and eGFR. E/N may differ across outcomes due to outcome‐specific eligibility and missing data on covariates/outcomes. No diabetes: FPG < 7 mmol/L and 2‐h PG < 11 mmol/L among participants without diagnosed diabetes. Confirmed undiagnosed diabetes: elevated levels of both FPG (≥ 7 mmol/L) and 2‐h PG (≥ 11 mmol/L) among participants without diagnosed diabetes. Isolated elevation of FPG: elevated levels of FPG (≥ 7 mmol/L) and 2‐h PG (< 11 mmol/L), among participants without diagnosed diabetes. Isolated elevation of 2‐h PG: elevated levels of 2‐h PG (≥ 11 mmol/L) and FPG (< 7 mmol/L), among participants without diagnosed diabetes. Incident treated diabetes: The *p* value of HR comparison of confirmed undiagnosed diabetes to isolated elevation of FPG was 0.001, applying Wald test. The *p* value of HR comparison of confirmed undiagnosed diabetes to isolated elevation of 2‐h PG was < 0.001, applying the Wald test. The *p* value for the comparison of HR between isolated elevation of FPG and isolated elevation of 2‐h PG, based on the Wald test, was 0.37. Incident first CVD: The *p* value of HR comparison of confirmed undiagnosed diabetes to isolated elevation of FPG was 0.06, applying Wald test. Incident hard CVD: The *p* value of HR comparison of confirmed undiagnosed diabetes to isolated elevation of FPG was 0.44, applying Wald test. Boldfaced hazard ratios (HRs) indicate statistically significant associations (*p* < 0.05).

Abbreviations: CI, confidence interval; CKD, chronic kidney disease; CVD, cardiovascular disease; E, events; eGFR, estimated glomerular filtration rate; HDL‐C, high‐density lipoprotein cholesterol; HR, hazard ratio; N, number; SBP, systolic blood pressure.

Compared with individuals without diabetes, participants with isolated elevation of FPG and those with isolated elevation of 2‐h PG had a significantly higher risk of developing treated diabetes, with HRs of 9.00 (95% CI: 6.26–13.00) and 7.40 (95% CI: 5.92–9.24), respectively.

The association between isolated elevation of FPG and 2‐h PG and incident hypertension was statistically significant only in Model 1. Those with isolated elevation of FPG had an HR of 2.12 (95% CI: 1.27–3.52), whereas individuals with isolated elevation of 2‐h PG had an HR of 1.52 (95% CI: 1.17–1.97).

Isolated elevation of FPG was significantly associated with an increased risk of both incident first CVD and incident hard CVD, with HRs of 2.55 (95% CI: 1.50–4.31, *p* = 0.001) and 2.43 (95% CI: 1.06–6.00, *p* = 0.04), respectively. In contrast, isolated elevation of 2‐h PG was not significantly associated with either outcome, with HRs of 1.23 (95% CI: 0.82–1.84, *p* = 0.30) and 1.00 (95% CI: 0.52–1.89, *p* = 0.99), respectively. Furthermore, neither isolated elevation of FPG nor isolated elevation of 2‐h PG was significantly associated with incident CKD or all‐cause mortality.

Results of the sensitivity analyses using imputed data are presented in Tables S1 and S2. Overall, the findings were largely consistent with those from the complete‐case analysis. Notably, the association between isolated elevation of FPG and incident hard CVD was attenuated and no longer statistically significant (Table S2).

## 4. Discussion

This study investigated the prognostic implications of confirmed and unconfirmed undiagnosed diabetes in a prospective cohort with approximately 10 years of follow‐up in the MENA region, focusing on long‐term outcomes including hypertension, CKD, CVD, and all‐cause mortality. Individuals with confirmed undiagnosed diabetes, defined by concurrent elevations in FPG and 2‐h PG at baseline, had substantially higher risks of progression to treated diabetes, as well as incident CKD and first CVD events, including hard CVD. Unconfirmed undiagnosed diabetes, defined by an elevation in either FPG or 2‐h PG alone, was associated with an approximately 50% higher risk of a first CVD event, comparable with that observed in confirmed diagnosed diabetes. However, the risk of progression to treated diabetes in this group was lower than in those with confirmed undiagnosed diabetes. Notably, elevated FPG alone, but not elevated 2‐h PG, was associated with risks of first and hard CVD events similar to those observed in confirmed undiagnosed diabetes.

Undiagnosed diabetes remains common globally and in the MENA region. In 2021, the International Diabetes Federation (IDF) reported that 45% of adults worldwide and 38% in the MENA region were unaware of their diabetes status [6]. Several studies conducted across various countries in the MENA region revealed a wide range of undiagnosed diabetes prevalence, from 7.7% to 57.8% [18–20]. National studies in Iran showed a significant decrease in the prevalence of undiagnosed diabetes, dropping from 45.7% to 24.7% between 2005 and 2011, primarily due to increased awareness of the disease [21]. Of note, all the cited studies have assessed the prevalence of undiagnosed diabetes by relying on single glucose diagnostic tests. In that context, our study extends the literature by examining the long‐term prognostic implications of confirmed versus unconfirmed undiagnosed diabetes defined by FPG and 2‐h PG.

Current clinical guidelines emphasize the need for a confirmatory approach before initiating pharmacological treatment for diabetes [5]. Confirming diabetes with a repeat test at a second‐time point can be logistically challenging and costly and may delay patient care. In this regard, Selvin et al. assessed the prognostic value of two definitions of undiagnosed diabetes, confirmed, using elevated FPG and HbA1c simultaneously, and unconfirmed, elevated by only one of the mentioned criteria, in the ARIC study. Compared with unconfirmed undiagnosed diabetes, confirmed undiagnosed diabetes showed a significantly stronger association with the development of diagnosed diabetes, CVD, and peripheral artery disease. However, both conditions were similarly associated with incident CKD and all‐cause mortality events. This study highlights that even one elevated marker is associated with a higher risk of progressing to overt diabetes and its complications [9]. In our data analysis, applying FPG and 2‐h PG criteria at the same time, we found confirmed undiagnosed diabetes was significantly associated with incident treated diabetes, CKD, CVD, and hard CVD events; however, unconfirmed undiagnosed diabetes was associated with incident treated diabetes, incident hypertension, and incident CVD events.

Based on a 12.1‐year follow‐up of 5573 participants in the Whitehall II study in the United Kingdom, Tabák et al. evaluated the percentage of diabetes cases diagnosed through an OGTT, whether by abnormal FPG, 2‐h PG, or both, that could also be confirmed using HbA1c levels. Their analysis revealed that new HbA1c‐confirmed diabetes in individuals with OGTT‐diagnosed diabetes was associated with a 53% increased risk for CVD and a 69% increased risk for CKD. Additionally, the authors found that individuals with diabetes diagnosed by OGTT but normal HbA1c levels have a risk of CVD and CKD comparable with that of the nondiabetic population [10]. In contrast, we found that undiagnosed diabetes ascertained by glucose determination—whether elevated FPG or 2‐h PG—when confirmed by another concurrent glucose determination, was associated with cardiovascular and renal outcomes.

The present study observed that unconfirmed undiagnosed diabetes was associated with a 50% higher risk for incident CVD events. This association was stronger for elevated FPG than for elevated 2‐h PG. The elevated FPG was also accompanied by about 140% higher risk for hard CVD. Regarding the association between elevated FPG and CVD risk, a study by Kaneko et al. explored the link between elevated FPG levels and CVD risk in Japanese young adults using a nationwide database analysis. The researchers examined medical records of 1,180,062 individuals aged 20–49 with no prior CVD history. Over a mean follow‐up period of 1201 days, the study found that individuals with elevated FPG had a 31%–109% increased risk of cardiovascular outcomes [22]. Moreover, a meta‐analysis of five prospective cohorts included in the Diabetes Epidemiology: Collaborative Analysis of Diagnostic Criteria in Asia (DECODA study group) revealed that unconfirmed undiagnosed diabetes, defined as elevated FPG or elevated 2‐h PG, was associated with an HR of 3.42 (95% CI: 2.23–5.23) with CVD mortality [23].

The current study showed that isolated FPG and 2‐h PG elevations at baseline were similarly associated with the risk of incident treated diabetes. FPG and 2‐h PG reflect different aspects of glucose regulation. FPG primarily assesses hepatic glucose production under fasting conditions, whereas 2‐h PG evaluates glucose disposal following a glucose load, reflecting both hepatic and peripheral insulin sensitivity [24]. Therefore, elevated FPG and 2‐h PG simultaneously had a higher risk for the development of treated diabetes [24, 25]. A study by the DECODA study group compared the performance of FPG and 2‐h PG in terms of diagnosis of T2DM across 11 population‐based studies in Asian countries [26]. The study indicated that FPG and 2‐h PG identify different groups of people. It concluded that, at the time of the study, when obesity was less common in the East Asian population, 2‐h PG was an essential diagnostic tool. Studies among the European population [25, 27, 28] found that relying on the FPG criteria was more likely to diagnose obese diabetic subjects. Still, the study reported that this was not the case among Asians, as less than 5% of the newly diagnosed diabetic individuals in the study population had a BMI of ≥ 30 kg/m^2^ [26]. In the current study, the mean BMI among newly diagnosed diabetes cases, whether confirmed or unconfirmed, was at the obesity level, which is consistent with a stronger association of elevated FPG with progression to treated diabetes. For surveillance purposes, relying on a single biomarker, predominantly FPG, tends to underestimate the prevalence of diabetes [2, 29]. This issue is even more pronounced in low‐ and middle‐income countries, where a significant portion of conditions like diabetes and hypertension often remain undiagnosed [30]. Despite these facts, our data suggest that an isolated elevated FPG in individuals not previously diagnosed as diabetic subjects could serve as a valuable prognostic marker for future clinical outcomes, particularly incident CVD events. These findings would be more meaningful when interpreted in the context of the MENA region, which bears a high burden of cardiometabolic risk factors and is predominantly composed of low‐ and middle‐income countries.

## 5. Strengths and Limitations

The current study has several strengths. First, its prospective population‐based cohort design includes a large sample size and a relatively long follow‐up period. Second, standardized measurements, such as data collected through validated questionnaires and laboratory assays, minimize the bias typically associated with self‐reported data. Lastly, to our knowledge, this is the first study to investigate the association between different phenotypes of undiagnosed diabetes (unconfirmed and confirmed) and the incidence of clinical outcomes in the MENA region, where cardiometabolic risk factors are highly prevalent [31].

Several limitations should be considered. First, statistical power may have been limited for outcomes such as incident hard CVD and all‐cause mortality, likely due to the relatively small number of events. Second, misclassification of baseline glycemic phenotypes is possible. Under the TLGS protocol, HbA1c was not measured, and classification relied on FPG and 2‐h PG from a single visit. As a result, some individuals with truly confirmed diabetes may have been categorized as “unconfirmed” when a corroborating test was unavailable. Evidence from the Whitehall II study shows that only about half of individuals with isolated fasting or 2‐h glucose abnormalities, but nearly all with both values abnormal, met HbA1c criteria for diabetes during follow‐up, and excess risks of CVD and incident CKD were confined to those with HbA1c‐confirmed diabetes [10]. In our cohort, the median FPG among individuals with confirmed undiagnosed diabetes (~8.5 mmol/L) corresponds to an estimated HbA1c of ~7.0%–7.5%, whereas the median FPG in the unconfirmed group (~6.5 mmol/L) corresponds to an HbA1c below 6.5% [32], suggesting limited overlap in true glycemic burden. Any remaining misclassification was likely nondifferential and may have attenuated group differences, although a risk gradient was still observed. Third, baseline diabetes status was determined cross‐sectionally from a single OGTT; therefore, the true onset and duration of hyperglycemia were unknown. This likely introduced a mix of recent and long‐standing undiagnosed cases, potentially biasing associations in either direction and limiting adjustment for disease duration or separation of lead‐time effects from true risk differences. Fourth, exact dates of treated diabetes onset were unavailable; event times were approximated as the midpoint between visits, assuming a uniform distribution, which may introduce bias in time‐to‐event estimates [33]. Fifth, as participants were drawn from metropolitan Tehran, generalizability to rural populations or other ethnic groups may be limited. Sixth, despite adjustment for multiple covariates, residual confounding, such as socioeconomic status, diet, disease duration or severity, measurement error, nonlinear associations, or other unmeasured factors, cannot be excluded. Finally, although concurrent abnormalities in FPG and 2‐h PG were associated with higher risks of future diabetes and vascular outcomes, their role in population screening requires further evaluation of feasibility, cost‐effectiveness, and clinical utility.

## 6. Conclusions

In this population‐based study, confirmed undiagnosed diabetes, defined by concurrent elevations in FPG and 2‐h PG levels, was associated with increased risks of treated diabetes, CKD, and incident first and hard CVD events. Compared with confirmed undiagnosed diabetes, the unconfirmed undiagnosed phenotype conferred a similar risk for incident first CVD but was accompanied by an approximately 50% lower risk of progression to treated diabetes. Finally, the excess risk of first and hard CVD events appeared to be driven primarily by isolated elevation of FPG rather than isolated elevation of 2‐h PG; notably, the CVD risk associated with isolated FPG elevation was comparable with that observed in confirmed undiagnosed diabetes.

**Nomenclature**
2‐h PG: 2‐hour post‐glucose
BMI: body mass index
CAD: coronary artery disease
CHD: coronary heart disease
CI: confidence interval
CKD: chronic kidney disease
CKD‐EPI: CKD Epidemiology Collaboration
CVD: cardiovascular disease
DALY: disability‐adjusted life years
DBP: diastolic blood pressure
DECODA: Diabetes Epidemiology: Collaborative Analysis of Diagnostic Criteria in Asia
DECODE: Diabetes Epidemiology: Collaborative analysis Of Diagnostic criteria in Europe
eGFR: estimated glomerular filtration rate
FPG: fasting plasma glucose
HbA1c: hemoglobin A1c
HDL‐C: high‐density lipoprotein cholesterol
HR: hazard ratio
MENA: Middle East and North Africa
NCD: noncommunicable diseases
OGTT: oral glucose tolerance test
SBP: systolic blood pressure
T2DM: type 2 diabetes mellitus
TG: triglycerides
TLGS: Tehran Lipid and Glucose Study

## Author Contributions

Navid Ebrahimi: conceptualization, methodology, writing—original draft, writing—review and editing. Samaneh Asgari: conceptualization, data curation, formal analysis, writing—review and editing. Maryam Tohidi: data curation, resources, writing—review and editing. Davood Khalili: supervision, writing—review and editing. Fereidoun Azizi: resources, supervision, writing—review and editing. Sepideh Besarat: writing—review and editing. Farzad Hadaegh: conceptualization, project administration, supervision, writing—review and editing.

## Funding

No funding was received for this manuscript.

## Ethics Statement

Ethical approval of this study has been certified by the central institutional review board (IRB) of the Research Institute for Endocrine Sciences (RIES), Shahid Beheshti University of Medical Sciences, Tehran, Iran. The outlined principles in the Declaration of Helsinki have been respected in this study. The current study received approval from the IRB of the RIES at Shahid Beheshti University of Medical Sciences, with the ethics Code Number IR.SBMU.ENDOCRINE.REC.1404.041. Written informed consent was obtained from all participants.

## Consent

All authors have declared their consent for publication.

## Conflicts of Interest

The authors declare no conflicts of interest.

## Supporting information


**Supporting Information 1** Additional supporting information can be found online in the Supporting Information section. Table S1: Multivariable hazard ratios (HRs) and 95% confidence intervals (CIs) for clinical outcomes by baseline diabetes phenotype in the TLGS (2002–2005): Imputed dataset. Table S2: Multivariable hazard ratios (HRs) and 95% confidence intervals (CIs) for clinical outcomes by isolated elevation of FPG, isolated elevation of 2‐h PG, and confirmed versus unconfirmed undiagnosed diabetes in the TLGS (2002–2005): Imputed dataset. Figure S1: Flowchart of the study population based on the imputed dataset in the Tehran Lipid and Glucose Study (2002–2020).

## Data Availability

The used datasets for analysis of the current study are available from the corresponding author upon reasonable request.
