# Supplementary material for: Long‐Term Health Outcomes in Individuals With Confirmed Versus Unconfirmed Undiagnosed Diabetes Based on Oral Glucose Tolerance Test: Findings From the Tehran Lipid and Glucose Study
Source: J Diabetes Res. 2026 Jul 8;2026:4228661. doi: 10.1155/jdr/4228661 (PMC13343168; doi:10.1155/jdr/4228661)
Supplement: Supplementary file 1 — Supporting Information 1 Additional supporting information can be found online in the Supporting Information section. Table S1: Multivariable hazard ratios (HRs) and 95% confidence intervals (CIs) for clinical outcomes by baseline diabetes phenotype in the TLGS (2002–2005): Imputed dataset. Table S2: Multivariable hazard ratios (HRs) and 95% confidence intervals (CIs) for clinical outcomes by isolated elevation of FPG, isolated elevation of 2‐h PG, and confirmed versus unconfirmed undiagnosed diabetes in the TLGS (2002–2005): Imputed dataset. Figure S1: Flowchart of the study population based on the imputed dataset in the Tehran Lipid and Glucose Study (2002–2020). [file JDR-2026-4228661-s001.pdf]

Long-term Health Outcomes in Individuals with Confirmed vs. Unconfirmed Undiagnosed Diabetes Based on Oral Glucose Tolerance Test: Findings from the Tehran Lipid and Glucose Study

Table S1. Multivariable hazard ratios (HRs) and 95% confidence intervals (CIs) for clinical outcomes by baseline diabetes phenotype in the TLGS (2002–2005): Imputed dataset

		Model 1		Model 2	
	**E/N**	**HR (95% CI)**	***P* value**	**HR (95% CI)**	***P* value**
**Incident treated diabetes**					
No diabetes	694/6167	Reference		Reference	
Unconfirmed undiagnosed	145/244	**8.45 (7.03-10.16)**	**<0.001**	**7.09 (5.88-8.55)**	**<0.001**
Confirmed undiagnosed	242/301	**20.81 (17.78-24.35)**	**<0.001**	**15.43 (13.11-18.17)**	**<0.001**
**Incident CKD**					
No diabetes	1811/5937	Reference		Reference	
Unconfirmed undiagnosed	97/225	**1.22 (1.00-1.50)**	**0.05**	1.12 (0.91-1.38)	0.27
Confirmed undiagnosed	159/280	**1.60 (1.36-1.88)**	**<0.001**	**1.47 (1.25-1.74)**	**<0.001**
**Incident hypertension**					
No diabetes	1878/4896	Reference		Reference	
Unconfirmed undiagnosed	81/137	**1.60 (1.28-2.00)**	**<0.001**	1.22 (0.98-1.53)	0.08
Confirmed undiagnosed	106/178	**1.57 (1.29-1.91)**	**<0.001**	1.13 (0.93-1.38)	0.22
**Incident first CVD**					
No diabetes	547/5767	Reference		Reference	
Unconfirmed undiagnosed	44/207	**1.76 (1.29-2.39)**	**<0.001**	**1.46 (1.06-2.00)**	**0.02**
Confirmed undiagnosed	63/267	**1.99 (1.53-2.59)**	**<0.001**	**1.58 (1.21-2.06)**	**0.001**
**Incident hard CVD**					
No diabetes	230/5767	Reference		Reference	
Unconfirmed undiagnosed	19/207	**1.66 (1.04-2.65)**	**0.035**	1.33 (0.82-2.14)	0.24
Confirmed undiagnosed	30/267	**2.05 (1.40-3.00)**	**<0.001**	**1.67 (1.13-2.47)**	**0.01**
**All-cause mortality**					
No diabetes	303/5767	Reference		Reference	
Unconfirmed undiagnosed	21/207	1.33 (0.85-2.07)	0.21	1.24 (0.78-1.92)	0.37
Confirmed undiagnosed	31/267	**1.45 (1.00-2.09)**	**0.05**	1.42 (0.97-2.07)	0.07

Abbreviations: HR: hazard ratio; CI: confidence interval; TLGS: Tehran Lipid and Glucose Study; E: events; N: number; CVD: cardiovascular disease; CKD: Chronic kidney disease; eGFR: estimated glomerular filtration rate; HDL-C: high-density lipoprotein cholesterol.

Model 1: adjusted for age, sex
Model 2; adjusted for age, sex, BMI, family history of diabetes, education levels, current smoking, lipid medications, non-HDL-C, hypertension (except for incident hypertension), SBP (only for incident hypertension), eGFR.

Definitions:
- Known diabetes included individuals using glucose-lowering medication.
- No diabetes: FPG <7 mmol/L and 2h-PG <11 mmol/L among participants without diagnosed diabetes.
- Confirmed undiagnosed diabetes: elevated levels of both FPG (≥7 mmol/L) and 2h-PG (≥11 mmol/L) among participants without diagnosed diabetes.
- Unconfirmed undiagnosed diabetes: elevated levels of FPG (≥7 mmol/L) or 2h-PG (≥11 mmol/L), but not both, among participants without diagnosed diabetes.

Long-term Health Outcomes in Individuals with Confirmed vs. Unconfirmed Undiagnosed Diabetes Based on Oral Glucose Tolerance Test: Findings from the Tehran Lipid and Glucose Study

Table S2. Multivariable hazard ratios (HRs) and 95% confidence intervals (CIs) for clinical outcomes by isolated elevation of FPG, isolated elevation of 2-h PG, and confirmed vs. unconfirmed undiagnosed diabetes in the TLGS (2002–2005): Imputed dataset

		Model 1		Model 2	
	**E/N**	**HR (95% CI)**	***P* value**	**HR (95% CI)**	***P* value**
**Incident Treated diabetes**					
No diabetes	694/6167	Reference		Reference	
Confirmed undiagnosed	242/301	**20.81 (17.78-24.35)**	**<0.001**	**15.47 (13.14-18.21)**	**<0.001**
Isolated elevation of FPG	34/49	**11.14 (7.87-15.76)**	**<0.001**	**8.00 (5.61-11.41)**	**<0.001**
Isolated elevation of 2-h PG	111/195	**7.86 (6.40-9.65)**	**<0.001**	**6.86 (5.588.44)**	**<0.001**
**Incident CKD**					
No diabetes	1811/5937	Reference		Reference	
Confirmed undiagnosed	159/280	**1.56 (1.36-1.88)**	**<0.001**	**1.47 (1.25-1.74)**	**<0.001**
Isolated elevation of FPG	22/45	**1.55 (1.02-2.36)**	**0.046**	1.35 (0.88-2.06)	0.16
Isolated elevation of 2-h PG	75/180	1.15 (0.91-1.45)	0.23	1.07 (0.85-1.35)	0.56
**Incident Hypertension**					
No diabetes	1878/4896	Reference		Reference	
Confirmed undiagnosed	106/178	**1.57 (1.29-1.91)**	**<0.001**	1.13 (0.93-1.38)	0.22
Isolated elevation of FPG	16/28	**1.95 (1.19-3.19)**	**0.01**	1.62 (0.98-2.65)	0.06
Isolated elevation of 2-h PG	65/109	**1.53 (1.19-1.96)**	**0.001**	1.15 (0.90-1.48)	0.26
**Incident First CVD**					
No diabetes	547/5767	Reference		Reference	
Confirmed undiagnosed	63/267	**1.99 (1.53-2.59)**	**<0.001**	**1.58 (1.21-2.06)**	**0.001**
Isolated elevation of FPG	14/43	**2.89 (1.69-4.88)**	**<0.001**	**1.82 (1.05-3.14)**	**0.03**
Isolated elevation of 2-h PG	30/164	**1.48 (1.03-2.15)**	**0.035**	1.34 (0.92-1.94)	0.12
**Incident Hard CVD**					
No diabetes	230/5767	Reference		Reference	
Confirmed undiagnosed	30/267	**2.05 (1.40-3.00)**	**<0.001**	**1.67 (1.13-2.47)**	**0.01**
Isolated elevation of FPG	6/43	**2.90 (1.29-6.53)**	**0.01**	1.96 (0.85-4.49)	0.11
Isolated elevation of 2-h PG	13/164	1.38 (0.79-2.42)	0.26	1.16 (0.66-2.04)	0.60
**All-cause mortality**					
No diabetes	303/5767	Reference		Reference	
Confirmed undiagnosed	31/267	**1.45 (1.00-2.09)**	**0.05**	1.42 (0.97-2.07)	0.07
Isolated elevation of FPG	4/43	1.39 (0.52-3.74)	0.51	1.23 (0.45-3.35)	0.68
Isolated elevation of 2-h PG	17/164	1.31 (0.81-2.14)	0.27	1.22 (0.75-2.01)	0.42

Abbreviations: HR: hazard ratio; CI: confidence interval; TLGS: Tehran Lipid and Glucose Study; E: events; N: number; CVD: cardiovascular disease; CKD: Chronic kidney disease; eGFR: estimated glomerular filtration rate; HDL-C: high-density lipoprotein cholesterol; SBP: systolic blood pressure.

Model 1: adjusted for age, sex
Model 2; adjusted for age, sex, BMI, family history of diabetes, education levels, current smoking, lipid medications, non-HDL-C, hypertension (except for incident hypertension), SBP (only for incident hypertension), eGFR.

Definitions:
- No diabetes: FPG <7 mmol/L and 2h-PG <11 mmol/L among participants without diagnosed diabetes.
- Confirmed undiagnosed diabetes: elevated levels of both FPG (≥7 mmol/L) and 2-h PG (≥11 mmol/L) among participants without diagnosed diabetes.
- Isolated elevation of FPG: elevated levels of FPG (≥7 mmol/L) and 2h-PG (<11 mmol/L), among participants without diagnosed diabetes.
- Isolated elevation of 2-h PG: elevated levels of 2-h PG (≥11 mmol/L) and FPG (<7 mmol/L), among participants without diagnosed diabetes.

Long-term Health Outcomes in Individuals with Confirmed vs. Unconfirmed Undiagnosed Diabetes Based on Oral Glucose Tolerance Test: Findings from the Tehran Lipid and Glucose Study

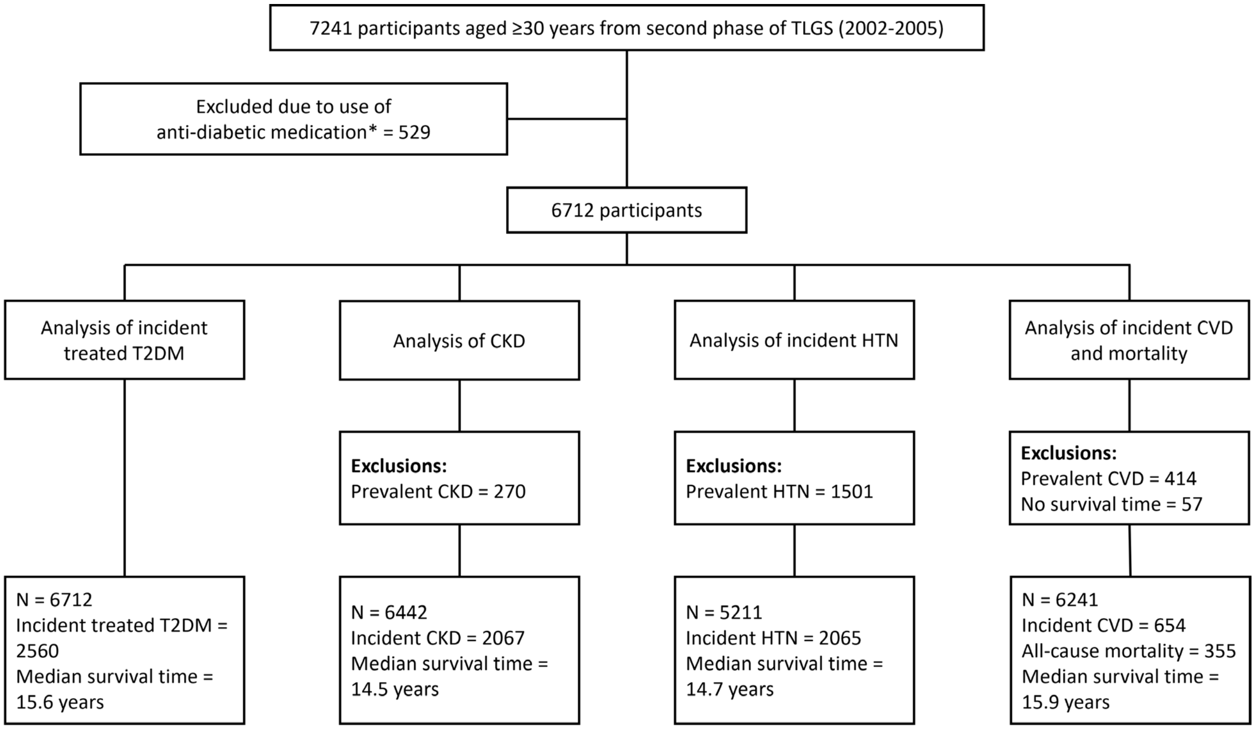

Figure S1. **Flowchart of the study population based on the imputed dataset in the Tehran Lipid and Glucose Study (2002–2020).**

* Participants using diabetes medications were excluded from the final analysis due to their small number.
